# Anatomical Characters of Asiatic Cholera

**Published:** 1835

**Authors:** 


### IX.—Anatomical Characters of Asiatic Cholera.

*On the Anatomical Characters of Asiatic Cholera, with Remarks on the Structure of the Mucous Coat of the Alimentary Canal. By W. E. Horner, M. D., Professor of Anatomy in the University of Pennsylvania.**

Notwithstanding the vast amount that has been written in relation to the Asiatic cholera, since the first introduction of this disease into Europe and America, it cannot be denied that our knowledge respecting it is still extremely vague and unsatisfactory, being in fact but little better than that of the world in general. The phenomena by which it is characterized, it is true, have been ably pourtrayed both by our own and foreign writers; but, with the exception of the generally

*American Journal of the Medical Sciences, No. xxxi., May, 1835.

admitted fact, that the disease is seated in the alimentary canal, little is known, with any certainty, as to the nature of the anatomical lesions to which it gives rise. Under these circumstances every physician, who has at heart the interests of his profession and the well-being of his patients, must hail with pleasure any effort, on the part of its members, that may have a tendency to throw light—even if it be but a feeble ray—upon the pathology of this singular disorder.

Amongst those who have been most conspicuous for their researches into the nature of Asiatic cholera, none, it may be truly said, hold a more distinguished rank than the author of the paper, the title of which stands at the head of this article. Possessing a rare amount of anatomical knowledge, and commanding opportunities of no ordinary kind, there is probably not an individual in the whole of our profession, that is so well qualified for making investigations of this description as Dr. Horner. The great interest which some of his essays on pathological anatomy have excited, both in this country and in Europe, sufficiently attests their value, and whilst it must prove highly flattering to the author, it cannot fail to stimulate him to further exertion in this extremely useful but hitherto too much neglected department of science.

The object of the essay before us seems, if I may so express myself, to be almost exclusively of a pathological nature, being intended to prove that in Asiatic cholera, the gastro-enteric canal exhibits certain anatomical lesions, which have hitherto escaped the attention of physicians. These morbid appearances may be thus stated:—1. An abundant vesicular eruption, which, extending throughout the whole alimentary canal, is perfectly distinct from the enlargement of the villi and muciparous follicles. 2. A lining membrane of coagulated lymph, similar in its texture and mode of adhesion to the false membrane of croup, and existing in the small intestines at least, if not also in the stomach and colon. 3. Vascular derangements and phenomena which are confined almost entirely, if not exclusively, to the venous system. 4. An exfoliation of the epidermic and venous lining of the intestinal canal, by which the extremities of the venous system are denuded, and left patulous.

The novelty of some of these positions, especially of the third and fourth, the latter of which assumes the existence of an epidermis in a part not generally admitted, has induced Dr. Horner to give a short exposition of the structure of the mucous coat of the alimentary canal, founded upon personal observation. His account occupies upwards of four closely printed pages, and it is to it, more particularly, that we wish to direct the attention of our readers.

> "The mucous coat of the alimentary canal, in a healthy state, and successfully injected, appears to consist almost entirely of a cribriform intertexture of veins; these veins being commonly empty at death, present themselves as a soft spongy structure, which give rise to the ordinary description of its sensible condition as a velvety layer. The most minute injection of the arteries scarcely makes itself visible among these veins when they are properly injected, a straggling branch only here and there exhibiting itself. The arboresence of the arteries is confined to the level beneath the venous intertexture, and is there developed to an extreme degree of minuteness, being intermixed with corresponding venous ramuscles, generally larger and more numerous than the arteries themselves. The fine venous trunks of this deeper layer have their originating extremities bent vertically towards the cavity of the gut, and by that means receive the blood of the first venous intertexture or layer, as the petrous sinuses join the cavernous, or the veins of the penis arise from its spongy structure." p. 60.

The superficial venous intertexture, as it is called by our author, forms, if we understand him correctly, a distinct layer, the meshes of which, being exceedingly minute, vary in a characteristic manner in the stomach, the small intestines, and the colon. This intertexture is said to have a very different arrangement from a common anastomosis, and in some places, as in the colon, to have the appearance of a plate of metal, pierced with innumerable apertures, closely bordering upon each other, and forming the mouths of so many mucous follicles. The edges of these openings are rounded off, and their depth does not extend beyond the superficial venous layer, being bounded below by the deep or arterio-venous layer, and by the subjacent cellular substance. The venous anastomosis here mentioned can be seen only after a successful injection, and for this purpose we may select either a vein or an artery; the latter being, however, objectionable, as it is necessary that the matter should be pushed into the veins, and whenever this is done we always lose, of course, the benefit which would result from a distinction of color between the two sets of vessels.

If this account is correct, it is obvious that the vessels of the gastro-enteric mucous membrane, are arranged very differently from what they have heretofore been supposed to be, and that there are two distinct sets of veins, the one deep-seated, and accompanying the arteries, the other superficial, and emptying into the former, in the same manner as one sinus of the dura mater does into another. In no organ of the body do we meet with a similar disposition of the veins, excepting in the liver, in which, as is well known, the vena portæ forms a distinct system by itself; whether the learned author really wishes to convey the meaning here expressed, it is by no means easy, owing to the extreme obscurity of his phraseology, to ascertain; if he does, the arrangement which he describes furnishes an anomaly of which no other anatomist before has ever dreamt; but that he really does not, may be gathered from his own statement. "Nothing short of an entirely successful injection will exhibit this venous anastomosis as described; and it may be seen either by injecting a vein or an artery, provided the injection passes from the artery into the veins."

Instead, therefore, of the two venous layers being distinct, as his language would lead us to infer, they are in reality most intimately connected, being directly continuous with each other, so that the superficial lamella may be injected from the deep, or the deep from the superficial. The fact is, that both sets of veins are derived from the same trunks; and the appearance described by Dr. Horner no doubt results merely from a peculiar interlacement of the vessels, not unlike that of the veins of the spermatie cord, or of those of the choroid coat of the eye.

That the gastro-enteric mucous membrane is most liberally supplied with blood-vessels, is a fact that has long since been familiar to anatomists; but that they present the peculiar arrangement pointed out by the author of the "Anatomical Characters of Cholera," may, I apprehend, be well doubted; at all events, further observations are required before his account can be received as in every respect correct. If we examine a portion of intestine, and carefully trace its vessels as they creep beneath the peritoneal covering, it will be found that, as they approach the sub-mucous cellular texture, they gradually decrease in size as they augment in number, until finally they resolve themselves into a beautiful capillary net-work. These ultimate ramifications, which are divided to an infinite degree of minuteness, being much smaller than the finest hair, are spread over the outer surface of the mucous corion, where they intersect each other in every conceivable direction, thus giving the intestine, when they are injected with size, colored with vermilion, the appearance of a piece of scarlet cloth. This vascular net-work, which may be compared in its arrangement and mode of formation to the vascular circles of the iris, was first demonstrated by Ruysch, so celebrated for the beauty of his anatomical preparations; and subsequently, in 1797, it was very admirably delineated by Bleuland, in his work on the minute structure of the alimentary canal.* The meshes or intervals of this net-work are occupied by numerous follicles and villi, which on being removed, give it the cribriform or seive-like aspect described by Dr. Horner. A very similar arrangement is observable in the pulmonary division of the mucous system, which derives its vessels chiefly from the bronchial arteries. These vessels, accompanying the most minute ramifications of the bronchial tubes, finally inosculate with the capilliaries of the pulmonary artery and veins, and when successfully injected, they impart a deep red tinge to the whole membrane.

The manner in which the capillary arteries of the mucous

---

*Vasculorem in Intestinorem Tenuium Tunis, etc., Descriptio Iconibus Illustrata. Trajecta ad Rhenum, 1797.

membrane terminate, has always been a subject of deep interest with the anatomist; but it can scarcely be said that our knowledge on this point, even at the present moment, amounts to any thing more than mere conjecture; at least such would seem to be the fact if we place any reliance upon what has been stated by our most distinguished authors. The thin sero-mucous fluid with which they are constantly moistened, induced Haller, and since his time, Sœmmering, Bichat, and others, to infer the existence of arteries with open mouths, or the presence of a set of serous vessels, communicating with the capilliary arteries, and forming what some writers on general anatomy have termed the exhalent system. No such vessels, however, there is reason to believe, exist; and hence the most philosophical explanation that can be offered, in the present state of our knowledge, respecting the termination of the capilliary arteries in question, is that they communicate directly with the capilliary or incipient veins; and in this opinion we are held out not only by the phenomena which are exhibited in examining the gastro-enteric mucous membrane in living animals, but likewise by what takes place in minute artificial injections.

Such, then, being the distribution of the vessels of the mucous system, the arrangement described by Dr. Horner as peculiar to the gastro-enteric division of it, cannot, it seems to us, be admitted; at least not until the researches of other anatomists shall have demonstrated his observations to be correct.

"Ordinary modes of examination give no evidence of the existence in the alimentary canal, from the cardiac orifice of the stomach to near the anus, of an epidermis; on the contrary, they rather lead to a belief of its being absent, in consequence of the softness, tenuity, and transparency of the mucous membrane; but that it is really present, may be proved by the following process:—Tear off the peritoneal coat—invert the part and inflate it to an emphysematous condition; the epidermis will then be raised as a very thin pellicle, and may be dried in that state." p. 61.

The existence of an epidermis or epithelium in the gastro-enteric mucous membrane, has been alternately admitted and denied by anatomists and physiologists, from the time of Haller until the present moment. The peculiar manner in which the œsophagus is inserted into the stomach, has induced many to believe that the epidermoid lining of that tube terminated at the cardiac extremity of that organ, precisely in the same manner as the corresponding covering of the vagina ends at the lips of the uterus. Haller and Bichat, admitting that it is impossible to demonstrate the epidermis of the alimentary canal, nevertheless maintain that we may infer its existence from the membraniform substances which are occasionally discharged from the bowels. This supposition, however, as has been justly observed by Beclard and J. F. Meckel, may be very easily controverted by the fact that all these excretions are the result of inflammatory action, one of the effects of which is an effusion of sero-albuminous matter, which, after having acquired a certain degree of consistency, is gradually detached from the free surface of the mucous tunic; being finally expelled in the form of a distinct membrane, not unlike that of croup.

Exudations of the kind here alluded to are by no means uncommon, either in the gastro-pulmonary or genito-urinary division of the mucous system. In the larynx, they have been observed by almost every modern author, especially by Cheyne, Home, and Field; in the œsophagus, by Bretonneau, and Albers; in the stomach, by Baillie, Howship, Villerme, Godman, and Andral; in the bladder, by Willis, Ruysch, and Morgagni; and in the uterus, by Hunter, Clark, and Denman. In all these different situations the membranous productions are of an analogous character, differing merely according to the peculiar organ in which they are developed. In every instance on record the formation of the membranous substance has been preceded or accompanied by some degree of inflammatory action. In the cases mentioned by Godman* and Villerme,† as having occurred in the stomach, it was connected with chronic gastritis; in those of Andral,‡ with fever; and

---

*Philadelphia Journal, 1825.
†Archives Generales, t. xiv., 1827.
‡Clinique Medicale, etc. Paris, 1823.

in that of Howship,* it was the consequence of swallowing hot water.

Taking into consideration, therefore, the fact that the expulsion of these membranes is always preceded or accompanied by inflammation, and the fact also that they are essentially different from the epidermis in their appearance and chemical composition, it is obvious that they cannot be adduced as affording legitimate proof of the existence of an epithelium in the gastro-enteric division of the mucous system. Did this membrane really exist here, it is reasonable to suppose that it could be displayed by the same process that it can in the mouth, œsophagus, or the vagina; but, so far as I know, this has never yet been done, the most delicate dissection, maceration, and boiling having alike failed to accomplish it.

The process recommended by Dr. Horner for displaying this mucous epithilium, is by no means satisfactory, as it would be utterly impossible thus to raise it, even if it existed, from the inner surface of the alimentary canal. Let us suppose, for instance, that the peritoneal coat of the bowel is removed, and that the air finds its way freely into the cellular texture connecting the mucous and muscular tunics, and what will be the result? To raise, as the learned professor imagines, a thin pellicle similar to the mucous epidermis in other situations? Unquestionably not: the only effect which it will have will be to break up the cellular tissue and thus separate the two contiguous lamellæ, elevating them in the form of small transparent vesicles, almost as prominent on one side as on the other. I do not now speak hypothetically, but from personal observation, founded upon a repetition of the experiment suggested by Dr. Horner.

"As this pellicle retains the air, we hence infer that it lines the follicles, and is uninterrupted by any porforations. This epidermis, if the part be previously injected perfectly, shows dots of injecting matter, but no arborescence if it be inflated up from the veins. In so doing the villi disappear, are in fact unfolded." p. 61.

The villi, then, it would seem, according to the notions of our author, owe their shape and consistence solely to this delicate epedermoid pellicle, and not, as we have been heretofore taught to believe, to the proper mucous corion, the very ground-work of the mucous texture. Now, the fact is, Dr. Horner, in speaking of this pellicle, constantly loses sight of this distinction, supposing, no doubt, that although the connecting cellular substance is destroyed by the process of in-

*Practical Remarks on Indigestion, London, 1825.

sufflation, the air only passes between the outer surface of the mucous corion and the contiguous surface of his pretended epidermis. But how, the question may be asked, does the air get there? There are but two ways by which we can account for it; either it must rupture the mucous corion, or it must pass around the muciparous follicles which are embedded in it. The former of these opinions is not at all probable, as, in the event upon which it is predicated, the epidermis itself would be most likely to be ruptured, and thus allow the air to escape; and as to the latter, it assumes what is by no means the case, that the mucous corion is pierced with innumerable apertures,corresponding to the muciparous follicles, and giving it a peculiar seive-like appearance.

On the whole I cannot but think that our author has committed a most serious error—a real anatomical blunder—in this part of his essay, and that he has mistaken for an epidermis nothing more nor less than the mucous corion itself! To show that this belief is not unfounded,let us refer for a moment to the structure of this fundamental portion of the gastro-enteric mucous membrane. Consisting of a soft, spongy substance, it is pervaded by blood-vessels, nerves, and lymphatics, and varies in its thickness in different parts of its extent, though in most places it may be said to be very thin. Presenting no sensible difference in the stomach and the duodenum, it becomes gradually thinner in the jejunum and colon, its tenuity being greatest in the ilium and the rectum. Its tenacity is every where proportionate to its thickness, excepting in the stomach, where it is much stronger than in the rest of the alimentary canal.

The apparent thickness of the corion depends very much upon its muciparous follicles and villi, and upon the cellular tissue which remains attached to it when it is pealed off from the muscular tunic. "Divested of these structures, and examined by itself, it may be described, according to M. Gendrin,* as consisting of an exceedingly thin, delicate pellicle, very vascular and diaphanous, which is stretched over a layer of follicles and papillæ, so as to give them both a complete covering, at the same time that it lines the interior of the former of these bodies. Beneath this pellicle is a stratum of permeable cellular texture, which not only occupies the intervals between the follicles, but at the same time connects the capilliary vessels, which enter into their substance, or which assist in forming the villi. When the follicles become shrivelled, as they sometimes do in protracted chronic diseases, the

*Histoire Anatomique des Inflam., t. i., p. 499.

villi shrink, the cellular texture partially disappears, and the mucous membrane itself becomes singularly attenuated and softened, owing to the fact that it is reduced to what it really is,—a *pellicle* spread over rarefied cellular tissue. Hence, it will be perceived that the thickness of the gastro-enteric mucous membrane, as it appears on first inspection, is altogether apparent, being in reality foreign to it."

"To demonstrate the structure of the gastro-enteric mucous membrane," continues Gendrin, "it is necessary to inflate a portion of intestine, care being taken previously to remove some of its other tunics, to invert it, and tie it at each extremity. In this manner the submucous cellular texture is rendered emphysematous, and the pellicle above alluded to is raised in the form of small but very distinct vesicles. If a small piece be then detached, and placed under a microscope, the pellicle will be seen to be pierced by a multitude of small, rounded apertures."* Is it not a most singular circumstance that Dr. Horner and M. Gendrin, the one writing in 1835, and the other in 1826, nearly ten years apart, should, by pursuing *precisely* the same method of investigation, arrive at such very opposite conclusions, the former considering this "pellicle" as a well-marked epidermis, the latter nothing more or less than the proper mucous corion, the fundamental structure of the mucous tissue? What a difference, reader, between the vision of an American and that of a Frenchman!

The villi, piles, or villosities, as they have been variously denominated, of the gastro-enteric mucous membrane, although seen by Azelli and others, were first accurately described, in 1721, by an anatomist of the name of Helvitius. These little bodies are extremely numerous in the small intestines, especially in the duodonum, but they are by no means wanting, as is asserted by our author, in the stomach and the colon; for in the pyloric half of the former of these organs and in the upper division of the latter, they always exist in considerable abundance. They are best seen by inverting a portion of bowel, and then immersing it in pure water, when they can be made to move gently amidst the fluid, in the form of minute processes projecting from the inner surface of the mucous lining. Their size varies in different parts of the alimentary canal, being much larger in the upper than in the lower portion of its extent. Their length, according to the measurements of Lieberkuhn† and Hildebrandt,‡ is scarcely the fifth

*Op. Cit., p. 500.
†De Fabrica et Act. Villos. Intest. Hom. Lugd. Bat., 1799.
‡Hildebrandt's Handbuch der Anatomie, 4ter Band, p. 275.

of a line; but according to J. F. Meckel,* they are considerably longer, being about the forty-eighth part of an inch, or the fourth of a line: when they are injected or rendered turgid, as they naturally are during digestion, their dimensions become almost double to what they are under other circumstances. They are arranged very compactly, and as there are about four thousand to every square inch, Meckel supposes that the whole number of them may be estimated at more than one million.†

The shape of these little bodies has long been an object of study with the anatomist; but as yet our knowledge in relation to this topic can scarcely be said to amount to any thing satisfactory. Helvitius,‡ Alfred Meckel,|| Dollinger,§ and Weber,¶ represent them as being flattened; Lieberkuhn** and Sheldon,†† as bulbous; Mascagni‡‡ and Sœmmering,|||| as conical, or of the shape of a mushroom; Hewson,§§ J. F. Meckel,¶¶ Rudolphi, and Hedwig,*** as cylindrical. The observations of Beclard on these little bodies are interesting, and deserving of notice, especially as they are founded on minute personal investigations. To appreciate the value of his remarks it is necessary to state that this anatomist employed an apparatus which was composed of a glass sphere, of small diameter, open on one-fourth of its surface, and of an operculum a little smaller than the opening, and of a thin layer of wax. The part to be examined was fixed on the wax with small pins, after which it was immersed in water, with the open sphere, which was then filled with that fluid, and placed on the operculum. The apparatus was then withdrawn, and the object to be examined was thus covered by a small lenticular mass of water, which augmented its diameter.

Examined in this way, Beclard asserts that the villi appear neither conical, nor cylindrical, neither tubular nor saccular, but in the shape of leaflets or minute plates, so closely aggre-

---

*Manual of Anatomy, vol. iii. p. 266.

†Op. cit. vol. iii., p. 266.

‡Mem. de l'Academie des Sciences, Paris, 1721, p. 302.

||Archive fur die Physiologie B. v., p. 163.

§Briefe an Sœmmering, 1828, p. 15.

¶Notes to Hildebrandt's Anatomie, vol. iv., p. 276.

**Op. cit.

††History of the Absorbent System, p. 36.

‡‡Vasorum Lymphaticorum, Corsheim, 1787.

||||Bau des Menschlichen Korpers, B. iii.

§§Experimental Inquiries, part ii., p. 175.

¶¶Manual of Anatomy, vol. 3, p. 266.

***Grundriss der Physiologie, B. ii. p. 209.

†††Disquisitio Ampullulorum, etc., Lipsic, 1789.

gated or set together as to convey the appearance of a luxuriantly tufted pile. Their shape varies according to the manner in which they are examined, and according to the region in which they are situated. Those of the pyloric half of the stomach and of the duodenum, are broader than they are long, and form little delicate plates; those of the jejunum, are long and narrow, constituting true piles or villosities; at the end of the ilium, they are laminar; and in the colon, they can scarcely be distinguished.*

These statements, it will be perceived, are extremely discordant, and they can only be reconciled, it seems to us, on the assumption that these little bodies vary not only in different animals, but also in different parts of the same animal. Thus, Hedwig states that they are cylindrical in the horse; conical in the dog; pyramidal in the mouse; and club-shaped in the pheasant; and it is not at all improbable, we think, that some of the anatomists whose names have been mentioned, made their observations on some of these animals; thus giving rise to the remarkable discrepancy by which they are characterized.

In regard to the intimate structure of the villi scarcely less discrepency prevails amongst authors than there does in relation to their shape. According to Lieberkuhn, each of these little bodies receives several minute arteries and veins, a lacteal vessel, and a nerve; the whole of them being united together by a very delicate cellular substance. The lacteal vessel he supposes to be dilated into a little vesicle or pouch, of the shape of an egg, the capacity of which he estimates at the fifth of a cubic line. The interior of the vesicle is soft and spongy, and upon its walls are ramified the arterial branches, the blood of which is taken up by corresponding veins, which finally terminate in a single trunk. On the apex of each villus is a small orifice, which he believed to be the mouth of the lacteal vessel.

Cruikshank, Hewson, and W. Hunter, on the contrary, deny the saccular expansion of the villi, and infer that the lacteals are ramified in the same manner as the blood-vessels. They assert, moreover, that, instead of containing but one orifice, as represented by Lieberkuhn, each pile presents as many as fifteen or twenty. These observations, again, are contradicted by the researches of Rudolphi, A. Meckel, and Treviranus. The former of these distinguished physiologists, who examined the villi in man and in a considerable number of animals, declares that he has never been able to perceive the orifice described by Lieberkuhn, much less those mentioned by Cruikshank, Hewson, and Hunter, notwithstanding the great care which he took to demonstrate them. The result of these investigations corresponds very accurately with the microscopical observations of Beclard. He describes the villi as being semi-transparent, smooth on the surface, and destitute alike of an ori-

*General Anatomy, p. 195.

fice and a saccular enlargement. Denying the existence of the vascular texture recognized by most other writers, he asserts that the only thing that can be seen in the gelatiniform substance, of which he supposes them to be composed, is a series of globules, of extreme delicacy, and arranged in linear order. The base of each villosity, however, he represents as being encircled by a net-work of blood-vessels and lymphatics, of great tenuity.*

The vascularity of the villi is extremely great, and may be beautifully displayed by forcing fine injecting matter or quicksilver into the vena portæ. If this be successfully effected, they will present, in the one case, a deep red, and in the other, a white argentine appearance. Under these circumstances they are rendered turgid, and greatly augmented in bulk. The same thing takes place, as has been already stated, during digestion, when they become gorged with chyle. If they be exposed to the air, in a living animal, and examined with a powerful microscope, they are observed speedily to assume a rose-colored aspect, and to present a vast number of the most delicate pores, from which are constantly oozing minute drops of a thin transparent liquid, which assists in moistening the inner surface of the mucous tunic. This fluid is very different from the viscid, ropy matter which fills the intervals between the villi, and which is discharged from the muciparous follicles. May not the presence of this serous fluid in the intestinal canal, and the rapidity with which it may be secreted, enable us to account for the profuse watery discharges which occur in Asiatic cholera, and in some species of chronic diarrhœa? M. Gendrin is inclined to think that the intestinal villi are not only endowed with the power of sucking in chyle—which is evidently their chief function—but that they are also organs of exhalation and venous absorption. He considers them, indeed, as so many capillary prolongations, enveloped in a fold of the cellulo-vascular pellicle lining the interior of the intestinal tube.† This opinion in regard to the structure of these bodies appears to me to be extremely plausible, certainly more so than any other with which I am acquainted.

"The superficial venous layer has great regularity in the ileum, and the conical villi stand out beautifully from its partitions, or, in equivalent language, from the divisions of the follicles. In the upper part of the small intestine the follicles are in equal number to what they are in the ileum; the regularity of their arrangement being interrupted by the long serpentine and oval villi; but invariably the same venous intertexture exists and forms in both, the chief bulk of the villi by passing into them.

In the stomach the *follicles* vary much in size, and there is an arrangement whereby many smaller ones are seen to open into the larger; on an average about two hundred and twenty-five are found upon every eighth of an inch square, which would give of course to an inch square sixty-four times that amount, or fourteen thousand four hundred follicles, and conceding the whole stomach to present an area of ninety inches, which is probably below the mark when this organ is moderately distended, as exhibited in the preparation upon which this calculation is founded, the entire number of follicles is *one million two hundred and ninety-six thousand.*

The greater uniformity of size of these follicles in the colon, and its even surface, enable us to count them with more certainty, and they appear to exist at the beginning of this gut at the rate of about four hundred for every eighth of an inch

*General Anatomy, p. 192.

†Histoire Anatomique des Inf., t. 1, p. 507.

square, and in the sigmoid flexure at about two hundred to the same area; they become, in fact, both smaller and less numerous in descending towards the anus. Admitting the entire area of the colon to be five hundred inches, and nineteen thousand two hundred of these follicles, on an average, to exist on every inch square, the aggregate number will be *nine millions six hundred thousand.*

Again, estimating the whole area of the mucous coat of the small intestines at fourteen hundred and forty inches, and allowing for interruptions occasioned by villi, about twenty-five thousand follicles are found upon every square inch, and the two numbers multiplied, produce *thirty-six millions.*

The entire number of follicles in the whole alimentary canal is, by the preceding estimates, forty-six millions, eight hundred and ninety-six thousand. I am very far from pretending to have counted them all, but have made an approximation to the actual number by observing sections of different portions of the same subject, and verifying the observations upon other subjects. The external surface of the cutis vera presents, as it were, in outline the same arrangement; the venous reticular intertexture appearing broader, not quite so perfect, and more shallow, and forming the papillæ; but as additional experiments are wanting it, may be passed over with this transient notice; perhaps, indeed, a more skilful hand in adopting the hint may perfect the details.

In the stomach, the largest of these follicles is about 1-98th of an inch in diameter, and the smallest about 1-490th. In the colon the largest is about 1-245th of an inch in diameter, and the smallest about 1-490th. In the small intestines their size varies in about the same ratio as in the colon, but they are much more irregular in shape, being scattered more in groups in consequence of the villi intervening; some of them penetrate obliquely towards the foundations of the villi, hence when examined from the exterior, their distribution is more regular, and they are seen lodged in the cellular coat of the gut.

I have endeavored to keep the estimate of the number of follicles below what other calculators would make it upon an observation of my preparations, and a fair measurement of the area of the alimentary canal, lest the number may seem excessive and incredible; I have therefore the most reasonable assurance of being within bounds on that point. I may now ask their use; is it to secrete or absorb? If they are simply secernents of mucous, the number, one would think, much greater than so limited a secretion requires—moreover, why is it that they become smaller and less numerous towards the lower end of the large intestine, where greater lubrication is required for hardened fæces; in addition, are not the glands of Brunner, [solitariæ,] and of Peyer, [agminatæ,] amply sufficient to furnish the required mucus? Again, after most sedulous observations upon the villi of all kinds, finely erected by my injections, and placed under most accurate, simple, and compound microscopes, I find invariably a polished reflecting surface, uninterrupted by foramina, either at their ends or sides, while many of these follicles are found passing obliquely into their bases. An excellent Wollaston's doublet, which makes the villi of the ileum appear an inch long, exhibits them with a polished translucent surface, without foramina, except where a villus from accident has been broken, a contingency readily recognized by one in the habit of viewing them. Finally, if the lacteal foramina of Lieberkuhn and others, do exist in fact, why is it that the raising of the intestinal epidermis by inflammation does not exhibit these foramina by the air escaping through them, but on the contrary, admits of a dried preparation in that state, the villi being completely effaced.

Taking into consideration these several objections to the theory of the follicles being secreting orifices, it appears to me that a better idea of their use is called for, which suggestion is submitted to the profession, with the hope that a more capable person will remove the difficulty by additional confirmation of preceding theories, or by the invention of a new one; for my own part I am much inclined to adopt the opinion of their absorbing faculties. It is generally conceded that the erectien and prehension of the Fallopian tube is produced by a vascular turgescence, in which the veins, from their number, must execute an important part; in like manner as these follicles are formed in the midst of veins, their orifices only become erect and patulous by the distention of these veins, and cannot be seen, especially in the small intestines, unless an injection has succeeded fully; but the erection of these veins during digestion puts the follicles in a similar condition; there is therefore some ground of inference, that the act of the Follopian tube in conveying a germ, and of a follicle in conveying into the thickness of an intestine congenial matter, may be analogous." pp. 61-4.

I have extracted this account of the size and number of the mucous follicles entire, as it forms decidedly the most interesting and valuable part of the professor's paper; in fact, the only part worthy of his pen. If his estimate be correct, it follows that these little bodies are infinitely larger and more numerous than has been heretofore imagined; but as all microscopical observations are liable to great deception, and, more especially, as the whole of these follicles can never be distinctly seen under any circumstances, so as to enable us to count them, it is not at all improbable that the author has fallen into error, and that his evaluation greatly transcends the natural limits. We leave it, however, for what it is worth, rather preferring to let the reader draw his own inferences than to forestall his opinion by any thing we might say of our own.

In the healthy state the mucous follicles are always recognized with great difficulty; but in certain pathological conditions, especially in chronic diarrhœa, cholera, and dropsy, they are rendered very evident. Being situated in the sub-mucous cellular substance, they are covered over by the proper mucous corion, and are of an irregularly oval, rounded or spheroidal shape, presenting each a small orifice leading to a blind or shut cavity. They occur either separately, or are arranged into groups, but in general they are so much flattened that they do not project sensibly upon the free surface of the membrane. In children these bodies are naturally more developed than in adults, and consequently are more easily recognized. In order to see them distinctly, the mucous lining must be thoroughly cleansed, and then held between the eye and a strong light, when they will appear like minute dots or points, of a white or greyish color, and with a central aperture, the periphery of which is of a deep grey: the difficulty of seeing these little bodies will always be lessened if the part to be examined be previously steeped in warm water or vinegar, as was first recommended by Willis, and since by Lauret and Laissagne. Their structure, as far as it can be ascertained, seems to be very simple, being composed entirely of a congeries of vessels, connected together by the most delicate cellular substance. Their interior cavity is perfectly smooth and uniform, and bedewed with a thin mucous fluid, which is seen constantly oozing from it.

With regard to the supposition of our author, that these little bodies possess absorbing powers, it is scarcely necessary to remark that it is altogether hypothetical and unfounded. The philosophy of this supposition is beautifully exhibited in the passage where he asks the question, whether the glands of Brunner and of Peyer are not amply sufficient to furnish the mucous which is required for lubricating the alimentary canal. The learned professor would thus evidently have us believe that these glands are essentially different from follicles, or if, as they really are, identical in structure, that they perform a different function!

We have now finished what we have to say in relation to our author's exposition of the mucous coat of the alimentary canal, founded on his own profound "personal observation," and shall conclude this article by one or two extracts, in which are detailed some of the more prominent lesions of the gastro-enteric mucous membrane resulting from Asiatic cholera.

"The mucous coat of the stomach and small intestines had a strong inflammatory tinge; the latter were filled with sero-fibrinous fluid, and were lined in almost their whole length by a layer of fibrine; this layer was about half an inch in thickness, and adhered to the mucous coat as a layer of fresh fibrine does to the pleura or peritoneum; that is to say, with a degree of tenacity permitting it to be rubbed off with the finger or floated in water, so as to exhibit both its adhesion to the intestine and its distinction from it. Having taken a piece of intestine home and hardened it in alcohol, the layer of fibrine was made manifest beyond controversy, and required to be separated with the end of a scalpel, exhibiting in that way its course uninterruptedly over the valvulæ conniventes, and down in the depressions between them. The difference marked in the thickness of this membrane was, that it began as a film in the duodenum and increased insensibly to half a line at the lower end of the ileum. At the suggestion of Dr. Physick I took a piece of the intestine, which had been preserved in alcohol, and after a maceration of a week, in a room the temperature of which was 70 degrees of Fahrenheit, the membranous character of the lining was still preserved." p. 68.

"In this case I made a minute injection of the pyloric end of the stomach, of some sections of the jejunum, and of the beginning of the colon and adjoining part of the ileum. The injection penetrated both arteries and veins with an almost inconceivable minuteness. In regard to the veins, when the parts were dried they opened on the internal surface of the bowels and stomach, as if excoriation had left their orifices bare. Hundreds also of cholera vesicles were found on the mucous coat of the jejunum jutting out from its surface in a spherical form so distinct as to leave no doubt of their being neither tumefied villi, follicles nor glands; they were especially numerous along the bases of the valvulæ conniventes. The superficial venous layer of the colon was entirely detached, except in a few places, and there it seemed like the skin of a locust just ready to fall off, it being so loose that the injecting matter had not passed into it. It there presented the cribiform appearance, being actually perforated from side to side by the sloughing process. No vesicles were found in the colon, and probably for the reason that they had been removed by the sloughing of its cuticle, and superficial venous layer; neither were vesicles found in the stomach, and probably for the same reason." p. 75.

The reader will perceive that in the latter of these cases, the "superficial venous layer was entirely detached, excepting in a few places, and there it seemed like the skin of a locust ready to drop off, it being so loose that the injecting matter would not pass into it;" and that, in the former, the small intestines were lined, in almost their whole length, by a layer of fibrin, no less than half a line thick, and firmly adherent to the mucous coat. These facts are interesting, but they are far from proving the existence of an epidermis in the gastro-entiric mucous membrane. All that they prove is, that, in Asiatic cholera, there is a high degree of inflammatory action going on, followed occasionally by a partial sloughing of the mucous corion, and the formation of a false membrane, similar in its texture to that of croup. As to the vesicles which Horner describes as occurring sometimes in the stomach and bowels in this disease, it is by no means easy to account for their origin, unless we suppose that, in consequence of the violence of the inflammation, the mucous membrane becomes detached, and raised from the muscular tunic by air or gas being generated between them.

Dr. Horner has promised to continue this subject in the next number of the "American Journal;" and as we deem his remarks extremely valuable, we anxiously wait for the appearance of his paper. By the way, the present number of this journal embraces an unusual amount of pathological matter, and we would especially refer to the "reports" of Doctors Kirkbride, Gerhard, and Pennock, as containing much valuable information. S. D. G.

